# Towards health equity: a framework for the application of proportionate universalism

**DOI:** 10.1186/s12939-015-0207-6

**Published:** 2015-09-15

**Authors:** Gemma Carey, Brad Crammond, Evelyne De Leeuw

**Affiliations:** National Centre for Epidemiology and Population Health, Australian National University, Canberra, Australia; Centre for Epidemiology and Preventive Medicine, Monash University, Monash, Australia; Centre for Health Equity Training Research and Evaluation CHETRE, University of New South Wales, Ingham Institute, Sydney, Australia

## Abstract

**Introduction:**

The finding that there is a social gradient in health has prompted considerable interest in public health circles. Recent influential works describing health inequities and their causes do not always argue cogently for a policy framework that would drive the most appropriate solutions differentially across the social gradient This paper aims to develop a practice heuristic for proportionate universalism.

**Methods:**

Through a review the proposed heuristic integrates evidence from welfare state and policy research, the literature on universal and targeted policy frameworks, and a multi-level governance approach that adopts the principle of subsidiarity.

**Results:**

The proposed heuristic provides a more-grained analysis of different policy approaches, integral for operationalizing the concept of proportionate universalism.

**Conclusion:**

The proposed framework would allow governments at all levels, social policy developers and bureaucrats, public health professionals and activists to consider the appropriateness of distinctive policy objectives across distinctive population needs within universal welfare state principles.

## Introduction and background

The finding that there is a social gradient in health has prompted considerable interest in public health circles. In particular, the notion that ‘*The “hardest to reach” are often the ones we need to reach most*’ [1] has attracted speculation regarding how to deliver health equity. The Strategic Review of Health Inequalities in England introduced the concept of ‘proportionate universalism’ to this debate [2], suggesting that health actions must be universal, not targeted, but with a scale and intensity that is proportionate to the level of disadvantage.

Since its introduction into the public health lexicon, interest in proportionate universalism has grown substantially. In the year to date, it has been discussed and cited in over 50 articles and books. While clearly popular as a principle, discussions of proportionate universalism currently remain too general to be useful for practice. That is, there is little to no guidance on how such an approach could be implemented by governments and policymakers, or acted upon by practitioners working to reduce health inequalities.

This is demonstrated by the varying interpretations of the meaning and reach of proportionate universalism. Canning and Browser [3] suggest that a proportionate response would see direct health interventions for the most disadvantaged. Birch, in contrast, equates it with a dose–response approach, where those at the bottom of the gradient require more ‘health action’ than those higher up [4]. It is not clear, however, that a proportionate universal approach would see higher doses of the same interventions, or whether a range of interventions, services and programs would be developed that would cater to differing needs across the gradient. Elsewhere, Michael Marmot’s descriptions of proportionate universalism appear to favour universal provisions, speaking out strongly against targeting:

We concluded that universalist policies were preferable to those targeted at specific groups for several reasons…. targeting implies labelling with all the attendant hazards of stigma,… Targeting only those at highest risk misses much of the problem ([5], p. 295).

‘Targeting’ within the Marmot Review is described in terms of a proportionate investment of resources into different social groups [2]. Yet, this approach would necessarily require degrees of targeting during implementation action, when deciding how those resources should be distributed and in what form. In other words, while a proportionate investment may be negotiated at higher levels within government, when it comes to ‘carrying out’ this investment in practice, decisions will be made about what form programs and interventions will take which will necessarily include some individuals and exclude others [6, 7].

In this paper we develop this debate by offering a framework to assist in thinking through how universal and/or targeted health equity action could be designed and implemented. Here, ‘health equity action’ refers to action on the social drivers of ill health and inequalities – such as education, housing, the built environment, employment and income. A more fine-grained analysis of principles and visions, along with their consequences, leads to an operational framework for proportionate universality that takes a governance approach. We anticipate that this framework will be useful for clarifying what policy change social determinants of health advocates hope to achieve, and to give practitioners a greater sense of where their efforts may fit within a proportionate universal approach.

To construct the framework, we draw on relevant literature in political science and social policy, in addition to a glossary of different principles that are applied when making decisions about health policy and action aimed at universal and targeted health programmes [6]. These are outlined in some detail first, before being integrated and related to one another in the second half of the paper to create a practice-oriented framework.

## Methods

This paper draws on an interdisciplinary literature review, incorporating public health knowledge on population health with theoretical discussions in the field of welfare studies. The results of the first part of this review are presented in form of a glossary, published elsewhere [6]. To create this, searches for peer-review literature were conducted in major databases: ProQuest, Sociological Abstracts, Web of Science, Social Sciences Citation Index, Academic Onefile, ScienceDirect, Expanded Academic, and EBSCO. Search terms included: universalism, policy targeting, and vulnerable groups. In addition, key theoretical texts were reviewed from within the welfare studies literature, sourced through a combination of the authors’ experience in this area and the reference lists of articles identified through the above search strategy. In doing so, the review took into account both empirical and theoretical/conceptual insights. In order to build on our glossary for the creation of the framework presented in this paper, we reviewed relevant literature on proportionate universalism (using the same search strategy as above) and reviewed relevant reports such as the Marmot Review. The authors then drew on their substantial backgrounds in politics and policy to connect these concepts with known governing principles (including ‘subsidiarity’ and ‘joined-up government’). The resulting heuristic is aimed at advancing debate in this area. However, jurisdictional responsibilities vary between countries and, as a result, will require refinement for particular settings.

## The many types of universalism and targeting

Comparative welfare state inquiry is profoundly concerned with the core cultural and policy beliefs that generate social policies either universally available to all, or focused on categorical entitlements [8]. This field of scholarship has become increasingly sophisticated, from a simple ‘state expenditures and allocations’ approach, through Esping-Andersen’s ‘decommodification’ approach *(‘the degree to which individuals or families can uphold a socially acceptable standard of living independently of market participation’*), to a comprehensive and interdisciplinary approach to political and gendered determinants of welfare state roles in maintaining – and reducing – (health) inequity [9–11]. Within this literature, different policy frameworks have been examined which fall under the broad categories of ‘universal’ and ‘targeted’. We propose that creating a framework for proportionate universalism that can guide practice requires this type of nuanced understanding of what operational forms universal and targeted policies can take.

### Universalism

There are two well-recognised ‘universal’ paradigms in policymaking: general universalism and specific universalism.

General universalism favours impartial determination of welfare recipients, as well as impartial allocation of benefits. Here, universalism refers to the degree of impartiality applied to the process of selecting individuals or groups deemed eligible for assistance, and also in the dispensing of this assistance [12]. These ‘flat-rate’ benefits are given to all, irrespective of citizenship, class, means or need [13]. Examples of general universalism include infectious disease control and sanitation. In comparative health systems research, ‘Beveridge’ systems (e.g., the 1948–2013 National Health Service in the United Kingdom) provide for universal health coverage and can be seen as a systems level approximation of the general universalism paradigm [14].

Specific universalism defends and extends social rights, as a way of achieving impartiality [15]. Social rights, such as the right to education, health care and so on, are considered important as a prerequisite for full participation in society. It supports free, universal availability of public services such as education and healthcare on the basis of citizenship (though it does not necessarily guarantee universal access) [15]. Universal public health care systems, such as those in Australia and Canada, are examples of specific universalism. Additionally, scholars such as Marshall have argued that people have a moral right to welfare in compensation for the inequality arising from modern society [15], ‘Bismarck’ systems could be deemed examples of specific universalism approaches as they provide universal coverage based on occupational class delineations (e.g., in Germany where health insurance, covering all, is organised through industrial relations) [14].

It is important to note that few policies are truly universal. In fact, many welfare states and policies which have been described as ‘universal’ exclude certain groups by virtue of viewing populations as homogenous [16]. This means that they are either not really universal at all, or in practice have been found to incorporate various degrees of targeting [13]. For example, much universalism in the post-war era ignored the needs of women and minority groups and catered predominately to white males [16]. Significant gaps have also been identified in the ‘universalist’ programs of the Nordic states, particularly in benefits for immigrants and guest workers [17]. Even properly conceived universalist policies may fail to be universal in practice [13]. The provision of sanitation is genuinely universal in conception but structural barriers, such as the remoteness of some communities in places like Australia and Canada, may impede universal implementation. Others have argued more strongly that universalism cannot truly exist in practice, as judgements must constantly be made in the delivery of services about who gets what, against a range of criteria [18] For example, within a universal health care system decisions are routinely made about which individuals require which services, based on a combination of priority and perceived need.

Proponents of universalism have been accused of confusing ‘impartiality’ with uniformity and ‘equality of treatment’ with ‘sameness of treatment’ regardless of different needs or ability to access services [6]. A superficial version of equal treatment could, for example, lead to people with disabilities being allocated an equal quantity of resources as those without a disability, ignoring their greater needs.

### Targeting

In practice, proportionate universalism must combine a degree of ‘selectivism’ within a universal framework, otherwise it will fail to flatten the social gradient. We contend that, in practice, this selectivism or tailoring of resources will necessitate a degree of targeting; while universalism is regarded as a precondition of equality, it does little to promote redistribution and ignores existing inequalities [19]. Although we agree with the Marmot Review that policy-targeting is rife with difficulty [7], we can begin to minimise targeting failures through nuanced understanding of differing forms of targeting and the principles that underpin them.

Selectivism refers to targeting or tailoring of services, policies or programs for different groups – that is, the ‘proportionate’ response required across the social gradient. Hence, selectivism refers to the provision of services and support to select social groups [20]. There is a long history of selectivism in industrialised countries, where special initiatives are targeted at different groups such as the short and long-term unemployed and single-income families [13, 21]. Selectivism can be broken down into two categories: negative and positive.

Negative selectivism targets the provision of services and assistance on the basis of individual means (i.e. using means testing), within a universal framework, such as the provision of low income health care cards [22]. Positive selectivism aims to provide additional services and resources for certain groups on the basis of needs (e.g. without means testing) [23]. In health care, community health services for refugees or specific indigenous health programs are examples of positive selectivism where targeted approaches (that sit alongside universal services) are needed to cater for highly specific needs.. Another example in the health policy realm are maternal and child health services. These are targeted in the sense that they are trying to capture one group (new mothers and babies), even though we might expect a large number of women to pass through that group, at any one time targeting is required to identify and involve them.

In practice, no complete distinction between universalism and selectivism is possible and instead the key policy task lies in finding a frame that allows for an appropriate integration of the two in the context of the particular dominant political gaze of what has variously been called ‘the statute’ or ‘polity’ [24].

Alongside these debates, scholars have debated the role of ‘particularism’ in public service provision. Particularism refers to differentiation on the supply side of interventions. Proponents argue that different standards are appropriate for individuals and groups in different circumstances, and that government policies and programs need to address differences between individuals on the basis of diversity of needs, moral frameworks and social expectations [25]. This should be done in a way that is empowering, suggesting that the state should not make authoritative decisions on behalf of individuals.

Purchaser-provider models in disability care are an example of particularism principles in action, where funds are given directly to individuals so that they may ‘purchase’ a service from providers, which meets their particular needs. Similar ‘market’ based approaches are used in education in the US and aged care in Australia [26, 27]. Particularism requires an appreciation of the different social identities of different groups (requiring investigation of values, wants, norms *and* needs) [12]. Proponents of these models argue that they ‘empower’ individuals to make choices about services and care, and promote a more client focused service from providers (e.g. one that caters better to the specific needs of an individual) [28]. Particularism, then, seems central to the goals of proportionate universalism, both on the grounds that it provides appropriate and effective services along the social gradient and places empowerment of both individuals and communities at the centre [29]. Moreover, particularist approaches have significant potential for overcoming the inverse care and prevention law [30].

## A framework for proportionate universalism

Differing forms of universalism and targeting can be combined in such a way as to maximise the strengths of each, while forming a cohesive whole [12]. Arguably, an appropriate balance can be struck which guarantees principles of equality and fairness (central to the social gradient approach), with the need to allow for diversity and difference (i.e. effective targeting for different social groups). The more nuanced discussion of universalism and targeting discussed above forms the basis for an effective framework for ‘proportionate universalism’ in practice, which offers a greater level of specificity than current discussions.

To flatten the social gradient, proportionate universalism needs to incorporate elements of both general and specific universalism (i.e. sanitation for all, other universal protections for citizens such as safe water and education). Targeting, within a proportionate universalism framework, would necessarily need to be based on principles of positive selectivism. While unclear in the literature whether proportionate responses should be organised around ‘means’ (i.e. income) or ‘needs’, the goal of providing appropriate supports for different social groups would be best supported by a focus on need. Countries which utilise means-testing tend to be more unequal and less successful at reducing poverty [31]. A framework for proportionate universalism must protect social rights against forms of targeting based solely on income [12], while embracing a more sophisticated conceptualisation of impartiality than that which underpins universalism. Rather than treating impartiality as uniformity (as is often the case in universalist approaches), it would provide additional resources to groups in order to offset structural disadvantages [12].

Finally, an effective approach would require a degree of particularism in the design of policies and programs. It is well established that ‘more of the same’ is rarely effective for different social groups. In fact, in some cases ‘more of the same’ increases rather than reduces health inequity; mass media behaviour change programmes and workplace smoking bans increase health inequity [32, 33, 34]. Lorenc et al. have strong indications that uniquely ‘downstream’ public health interventions most profoundly follow the ‘inverse prevention law’ [32].

Incorporating particularist principles would see differentiation in the nature and supply of interventions, policies and programs so that they are tailored to the specific needs of different social groups, whether on the basis of values, ethnicity or other criteria. Hence, a framework that can achieve the goal of flattening the social gradient would need to be based on the position that under particular circumstances, different standards need to be applied to individuals and groups to ensure their needs and structural disadvantages are adequately met.

These principles alone are limited in their capacity to direct action in a practical sense [12]. How, for example, should decisions be made regarding ‘cut’ points between universal and targeted policies, or under what circumstances is a particularism approach warranted? To address these questions, we apply the principle of subsidiarity – a principle that has long been applied to questions of social justice.

**Fig. 1:**
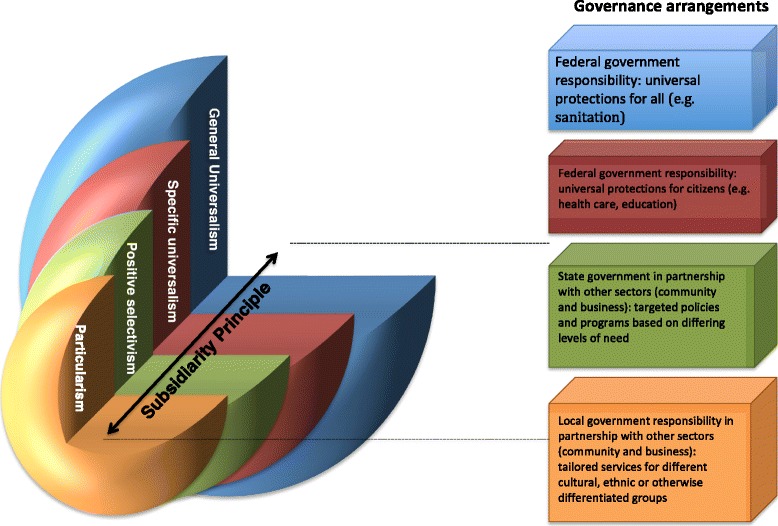
Proportionate Universalism Heuristic

Subsidiarity is both a principle of governance and a practical framework for solving social problems [35]. Subsidiarity seeks to ensure that decisions and actions are taken as closely as possible to citizens through a multi-layered system. Subsidiarity forms part of the governance architecture of the European Union and, more broadly, has guided reform in a range of policy changes in industrialised countries such as the US and Canada [35–37]. Subsidiarity is a “principled tendency toward solving problems at the local level and empowering individuals, families and voluntary associations to act more efficaciously in their own lives” (p116) [36]. We have chosen subsidiarity as a governance principle because it is broadly consistent in its goals with proportionate universalism (i.e. to empower individuals to shape decisions that impact their lives). In applying the principle of subsidiarity the framework outlined above, we can begin to delineate how such an approach could be operationalized, utilising differing levels and forms of governance (see Fig. 1).

Within our framework, decisions regarding what services need to be tailored to which individuals will be made by the level of governance closest to them; the rationale being that local government and non-government organisations, embedded in local communities, are more likely than federal governments to understand the needs of specific individuals and groups and how best to address them [38, 39].

At the other end of the scale, our framework draws attention to the critical role of federal governments in providing general and specific universalist policies, programs and services, for which they alone have the legitimacy, resources and authority to provide. Subsidiarity has been wrongly conflated with devolution, decentralisation and notions of ‘localism’ [40]. This has happened both in the US context were social functions are reallocated from higher to lower government agencies, or from government to non-government organisations [35] and under the recent UK Coalition government through its Localism Act [41].

A strong core of universal policies is critical to ensuring the health of populations [42]. The application of the subsidiarity principle would allow for a policy design where responsibilities at different governance levels complement and reinforce each other, creating more effective multi-level governance operations and policies [43]. This conceptualisation of subsidiarity therefore draws strongly on concepts of joined-up (or horizontal) government – where different administrative levels require integrated and complementary action to address social problems [44, 45]. Hence, where policy targeting failures appear (e.g. place-based targeting that fails to adequately capture disadvantaged groups [46]), the decision may be reached that a universal approach – implemented federally – is in fact the only way to overcome such failures. Similarly, where universal provision would be more cost-effective, subsidiarity would support federal action. For example, means-tested taxation systems, where those on low incomes receive proportionally greater benefits from those on higher incomes, have been found to cost more due to high administrative burden than simply offering universal tax benefits [47, 48]. Here, subsidiarity – and in a proportionate universalist approach – should support the latter.

As subsidiarity aims to empower individuals and voluntary associations, it places a focus on “fostering vitality of [these] mediating structures in society” ([36], p. 116). While decisions may appear to be devolved to lower levels of government, or non-government groups, in actual fact states must maintain a strong and vital role in ensuring that mediating structures (e.g. non-government organisations, charities or voluntary associations) are effective and do not encroach on the individual rights secured through universal policies. That is, governments remain responsible for the effectiveness of seemingly ‘devolved’ structures and must intervene accordingly. Indeed, it has been argued that subsidiarity should only be employed when doing so will foster, rather than impede, mediating structures [36].

While our proposed framework makes clear distinctions between the responsibilities of differing levels of governance to provide universal or targeted policies or interventions, subsidiarity refers to both vertical and horizontal governance. Vertically it ensures the appropriate allocation and exercise of ‘competence’; different levels of governance have different forms of legitimacy based on identity, knowledge, resources and legislation [36]. Vertical subsidiarity ensures that action is taken at the level with the greatest legitimacy to act to solve a given problem. However, for many policy areas this competence and legitimacy is shared, concurrent or overlapping (as, in fact, seen in the delivery of many healthcare systems) [36]. That is, there are a variety of ways in which social issues can be addressed which utilise different combinations of action at different levels. Hence, the application of the subsidiarity principle in practice may result in action at multiple governance levels. This means that some policies and interventions will be provided solely at one level of governance, while others will need partnership arrangements between different levels of the state, or between state and non-state actors. In industrialised countries, this is now the norm – with responsibilities increasingly fulfilled jointly by federal, state and non-state actors [39].

Perhaps the most well-known discourse on the ‘superiority’ of vertical versus horizontal approaches to health service (including public health and health promotion) delivery is found in the literature on Primary Health Care (PHC). Believers in ‘orthodox’ Declaration of Alma Ata PHC (a horizontal programme across governance systems designed and implemented as close as possible to communities in need – following the subsidiarity principle) have chastised vertical programmes dedicated to ‘single issues’ or worse, single diseases. Yet, such single disease vertical programmes have yielded considerable disease control effects (e.g., the eradication of smallpox) albeit by mobilising broader social investment [49, 50]). The binary between horizontal and vertical is untenable and synergies should be sought [51, 52]. These analyses also show that short-term interest group funding favours effectiveness of vertical programmes, whereas longer-term political commitment impacts on social goals of horizontal programmes. Hence, the horizontal/vertical dichotomy is a false dilemma [50]. Rather, supply-driven provision of highly cost-effective interventions ought to be coupled with the gradual implementation of multiple demand-driven public health interventions (which encourage resource sharing) [49, 50].

Identifying the right way to delineate different roles of actors at each level is complex. For example, what proportion of problems or solutions need to be shared in order to warrant joint responsibility? This complexity is further compounded by the fact that more often than not, these decisions are political rather than technical. The decentralisation of service provision and allocation decisions to local governments has, in some instances, been resited due to the additional administrative burdens this places on local authorities [53]. More equality requires more bureaucracy [12]. Similarly, decisions over what individuals are provided with what services or resources are inherently political, as well as technical. A value free assessment of need in political terms does not exist. Rather it rests on particular world views (liberal, social democrat, anarchist, etc.).

Finally, if implemented a national level the proposed framework would require careful monitoring, both of the system itself and population indicators. Ensuring the right balance between universal, targeted and particularist policies will be a constant challenge and area of refinement. However, experiences of balancing integration and diversity within the EU demonstrates that the types of balancing acts required to make subsidiarity work in practice are achievable, if not always straight forward. Within liberal welfare sate regimes – where inequalities are widest – there is a need to guard against residualism in welfare, where programs become increasingly targeted to the ‘poor’ [23]. Research has demonstrated that within such regimes, policy targeting has a powerful pull, due to both the historical norms of particular welfare states and the intuitive logic of such approaches [7].

## Conclusions

We observed that influential works describing health inequities and their causes - including the Marmot Review - do not always argue cogently for a policy framework that would drive the most appropriate solutions differentially across the social gradient. In response, we propose a proportionate universalism heuristic in which we integrate evidence from welfare state and social policy research, the literature on universal and targeted policy frameworks, and a multi-level governance approach that adopts the principle of subsidiarity.

Our framework would allow governments at all levels, social policy developers and bureaucrats, public health professionals and activists to consider the appropriateness of distinctive policy objectives across distinctive population needs within universal welfare state principles.
